# Acknowledgment of Reviewers

**DOI:** 10.2188/jea.JE20170322

**Published:** 2018-01-05

The following scientists assisted the journal by reviewing manuscripts during the period November 1, 2016 to October 31, 2017.

We gratefully acknowledge their critical evaluation and assistance in selecting articles for publication.

**Table:** 

Abe, Sarah K.
Aida, Jun
Akasaka, Hiroshi
Akter, Shamima
Almuneef, Maha
Ansai, Toshihiro
Arima, Hisatomi
Arima, Kazuhiko
Asao, Keiko
Asayama, Kei
Baba, Sachiko
Baik, Inkyung
Bao, Yan-Ping
Barinas-Mitchell, Emma
Bernhardsen, Guro P.
Budhathoki, Sanjeev
Cable, Noriko
Chei, Choy-Lye
Chihara, Izumi
Doering, Nora
Eguchi, Hidetaka
Elhakeem, Ahmed
Fowler, Helen
Fujita, Misuzu
Fujiyoshi, Akira
Fukuda, Yoshiharu
Fukuhara, Masayo
Fukui, Michiaki
Fukunaga, Ichiro
Gebel, Klaus
Goto, Atsushi
Goto, Aya
Goto, Yasuyuki
Graziose, Matthew M.
Guo, Jing
Gutin, Benard
Halligan, Joe
Hamazaki, Kei
Haruyama, Yasuo
Hata, Jun
Hayama-Terada, Mina
Heianza, Yoriko
Hidaka, Akihisa
Hirakawa, Yoichiro
Hisamatsu, Takashi
Hong, Seoah
Hori, Megumi
Hosono, Satoyo
Hsu, LI
Ichikawa, Masao
Ikeda, Fusao
Ikeda, Nayu
Imai, Teppei
Inada, Haruhiko
Inoue, Sachiko
Inoue, Shigeru
Inoue, Yosuke
Ishikawa, Hirofumi
Ishikawa, Shizukiyo
Isomura, Tatsuya
Itani, Osamu
Ito, Shinya
Ito, Yuri
Iwasaki, Masanori
Jee, Sun Ha
Kachi, Yuko
Kai, Yuko
Kajiyama, Hiroaki
Kakizaki, Masako
Kamada, Masamitsu
Kamiya, Yasuhiko
Kamo, Kenichi
Katanoda, Kota
Kato, Tuguhiko
Katsuda, Nobuyuki
Kawado, Miyuki
Kawaguchi, Takumi
Kawakami, Ryoko
Khosravi, Ardeshir
Kikuchi, Hiroyuki
Kim, Chan-Won
Kim, Dong-Jun
Kim, Hyun Ja
Kitabatake, Yoshinori
Kitajima, Tsuyoshi
Kitayuguchi, Jun
Kokubo, Yoshihiro
Kondo, Atsuo
Kotani, Kazuhiko
Koyama, Shihoko
Kubota, Yasuhiko
Kurumatani, Norio
Kuwahara, Keisuke
Lee, Soo-Kyung
Levin-Zamir, Diane
Li, Ming-Chieh
Lin, Yingsong
Lobo, Elena
Marklund, Matti
Matsuda, Tomohiro
Matsue, Yuya
Matsuyama, Ryota
Matsuyama, Yusuke
Matsuzaka, Masashi
Metoki, Hirohito
Michikawa, Takehiro
Mihara, Satoko
Minami, Ushio
Miyachi, Motohiko
Miyake, Taku
Mizoue, Tetsuya
Mori, Chisato
Morisaki, Naho
Muka, Taulant
Munakata, Masanori
Murakami, Keiko
Murakami, Yoshitaka
Muraki, Isao
Murayama, Hiroshi
Nagamine, Yuiko
Nagata, Chisato
Naito, Toru
Nakamura, Mieko
Nakata, Yoshio
Nakatochi, Masahiro
Nakayama, Meiho
Nanri, Hinako
Nishino, Yoshikazu
Noda, Hiroyuki
Noma, Hisashi
Oba, Shino
Obara, Taku
Ogawa, Kohei
Ohsawa, Masaki
Okumura, Yasuyuki
Ooki, Izumi
Otsuka, Rei
Ozaki, Tetsunori
Ozasa, Kotaro
Park, Sohyun
Petersen, Inge
Ranganathan, Rama
Risch, Harvey
Saika, Kumiko
Saito, Eiko
Saito, Isao
Saito, Junko
Saito, Masashige
Saito, Toshiyuki
Sakai, Rie
Sakurai, Masaru
Sakurai, Ryota
Sasai, Hiroyuki
Sasaki, Hideo
Satoh, Atsushi
Sawada, Norie
Sawada, Takayuki
Shaw, Benjamin
Shibata, Akiko
Shimazu, Taichi
Shimokawa, Mototsugu
Shimoya, Koichiro
Shin, Aesun
Shin, Sangah
Shinozaki, Tomohiro
Shirai, Kokoro
Shiroma, Eric
Skjærven, Rolv
Sloan, Robert
Sobue, Tomotaka
Sugiyama, Daisuke
Sugiyama, Hiromi
Sun, Xibin
Suzuki, Etsuji
Suzuki, Reiko
Suzuki, Sadao
Suzumori, Nobuhiro
Tabara, Yasuharu
Tabuchi, Takahiro
Tachimori, Hisateru
Taguri, Masataka
Takagi, Daisuke
Takahashi, Hideto
Takahashi, Kunihiko
Takahashi, Yoshimitsu
Takegami, Misa
Takeuchi, Ayano
Takimoto, Hidemi
Talaei, Mohammad
Tanaka, Akihiro
Tanaka, Chiaki
Tanaka, Junko
Tanaka, Sachiko
Taniguchi, Yu
Tanihara, Shinchi
Tanikawa, Chizu
Taurel, Anne-Frieda
Taylor, Mark
Togari, Akifumi
Tomata, Yasutake
Tomizuka, Taro
Tsubota, Megumi
Tsuboya, Toru
Tsuchiya, Naho
Tsuda, Toshihide
Tsugane, Shoichiro
Tsuji, Taishi
Tsunematsu-Nakahata, Noriko
Ueda, Kayo
Uemura, Hirokazu
Umesawa, Mitsumasa
Urayama, Kevin
Vo, Phuong
Wada, Keiko
Wakai, Kenji
Wallis, Belinda
Wander, Pandora
Wang, Chaochen
Watanabe, Shuichiro
Yamagishi, Kazumasa
Yamamoto, Seiichiro
Yamamoto, Tatsuo
Yamazaki, Shin
Yanagawa, Hiroshi
Yanagita, Masahiko
Yatsuya, Hiroshi
Yorifuji, Takashi
Yoshida, Daigo
Yoshida, Kazuki
Yoshinaga, Shinji
Zhang, Suxia

